# Meta-Analysis of ABCB1 3435C>T Polymorphism and Colorectal Cancer

**DOI:** 10.12669/pjms.295.3758

**Published:** 2013

**Authors:** Dan Zhang, Cun Wang, Zongguang Zhou

**Affiliations:** 1 Dan Zhang, Department of Gastrointestinal Surgery, West China Hospital, Sichuan University, No. 37 on Guoxue Xiang, Chengdu, Sichuan Province, China.; 2 Cun Wang, Department of Gastrointestinal Surgery, West China Hospital, Sichuan University, No. 37 on Guoxue Xiang, Chengdu, Sichuan Province, China.; 3 Zongguang Zhou Department of Gastrointestinal Surgery, West China Hospital, Sichuan University, No. 37 on Guoxue Xiang, Chengdu, Sichuan Province, China.

**Keywords:** Colorectal cancer, ABCB1, MDR1, P-gp, Polymorphism, Meta-analysis

## Abstract

***Objective:*** Many studies have focused on the association between the ABCB1 3435C>T polymorphism and colorectal cancer (CRC) risk. However, the results were conflicting. The aim of this meta-analysis is to evaluate the precise association between this polymorphism and CRC risk.

***Methods:*** We formally reviewed the literature at Pubmed, EMBASE and the Cochrane Library with the key words as follows: ABCB1/MDR1/P-glycoprotein, polymorphism, colorectal and cancer/neoplasm/tumor. This meta-analysis was assessed by Review manager 5.0. The fixed-effects model was used to pool the odds ratios (OR) with 95% confidence intervals (CI) for CRC risk.

***Results:*** There were 8 studies identified. The pooled OR with 95% CI of CC+CT versus TT genotype of the ABCB1 3435C>T polymorphism for CRC risk was 1.01 [0.90-1.13]. The sensitivity analysis further confirmed the result. Heterogeneity and publication bias were not observed in this meta-analysis.

***Conclusions:*** In summary, there was no significant association between the ABCB1 3435C>T polymorphism and CRC risk.

***Abbreviations used:*** the ATP-binding cassette, subfamily B, member 1 (ABCB1); multidrug resistance gene 1 (MDR1); P-glycoprotein (P-gp); colorectal cancer (CRC); single nucleotide polymorphisms (SNPs); odds ratio (OR); confidence interval (CI); Hardy-Weinberg equilibrium (HWE).

## INTRODUCTION

Colorectal cancer (CRC) is both the prevailing malignancy and one of the leading causes of cancer-related mortality in the Western World, with great impact on the life quality of affected persons.^1^^,^^2^ Every year, there are approximately 220,000 new cases of CRC diagnosed and 112,000 deaths in the European Union.^3^ Colorectal carcinogenesis is a complex, multistep and multifactorial progress which is caused by the interaction of many factors such as lifestyle, dietary and genetic susceptibility. In recent years, many studies have begun to recognize the importance of single nucleotide polymorphisms (SNPs) in genes that are involved in xenobiotic metabolism that might account for CRC risk. A number of common SNPs associated with CRC risk play a critical role in the development of CRC via modifying the expression of target genes that regulate cell behaviors.

The ATP-binding cassette, subfamily B, member 1 (ABCB1), also named multidrug resistance gene 1 (MDR1), is located on chromosome 7q21.1. It encodes P-glycoprotein (P-gp) which is a 170kDa transmembrane transporter that acts as an ATP-dependent efflux pump of xenobiotics and various chemotherapeutic drugs. P-gp is expressed in normal cells of various organs such as intestine, liver, kidney, brain, and placenta, which is involved in absorption and elimination of xenobiotics and drugs and hence, could be a risk factor of diseases. On the other hand, P-gp probably also plays a role in regulating cell death, differentiation, and proliferation, as well as in immune response.^4^ It is found that P-gp is highly expressed on the atypical surface of differentiated tubular structures in CRC and the expression of P-gp is associated with the progression of CRC.^5^^,^^6^ The ABCB1 gene is polymorphic and to date approximately 48 SNPs have been identified.^7^ SNPs probably change the functional expression of the ABCB1 gene so that predisposing to diseases.

The well-known ABCB1 gene polymorphism is 3435C>T polymorphism. The 3435C>T polymorphism in exon 26 is a synonymous C to T transformation, which encodes the amino acid isoleucine and probably affect the expression of ABCB1 gene and the function of P-gp in certain way.^8^ There were studies focused on the ABCB1 3435C>T polymorphism, which had influence on inflammatory bowel disease risk rather than breast cancer risk.^9^^,^^10^ For CRC, some studies have reported a link between ABCB1 3435C>T polymorphism and CRC risk,^11^^-^^14^ while others have reported conflicting results.^15^^-^^18^ Hence, whether the ABCB1 3435C>T polymorphism is associated with CRC risk still remains controversial.

To resolve the dispute, we have presented a meta-analysis of the association between the ABCB1 3435C>T polymorphism and CRC risk.

## METHODS

***Study Selection:*** Search was applied to the following electronic databases: the Cochrane Library (first quarter, 2013), Pubmed (1966 to February 2013) and EMBASE (1980 to February 2013). The following key words were used: “ABCB1 or MDR1 or P-glycoprotein”, “polymorphism”, “colorectal” and “cancer or neoplasm or tumor”. The research was conducted on human study, and non-English language studies were excluded. The reference lists of reviews and retrieved articles were hand searched at the same time. We did not consider abstracts, letters and unpublished studies. When more than one studies of the same population were identified, we included the study with the largest sample size. We reviewed abstracts of all citations and retrieved studies. For inclusion in this meta-analysis, the identified studies had to provide information on: (1) the number of CRC cases and controls studied; (2) the number of homozygous and heterozygous genotypes of ABCB1 3435C>T polymorphisms in cases and controls. Major reasons for exclusion of studies were: (1) no control; (2) duplicate; (3) no usable data reported.

***Data Extraction: ***All data were extracted independently by two reviewers (Dan Zhang and Cun Wang) according to the prespecified selection criteria. The following data were extracted: the first author’s surname, publication year, ethnicity, genotyping methods, number of cases and controls, number of genotyped cases and controls (CC, CT and TT genotypes of ABCB1 3435C>T polymorphism). Disagreement was resolved by discussion. If a consensus was not reached by discussion, a third party (Zongguang Zhou) was consulted to resolve the dispute.

***Statistic Analysis: ***The meta-analysis was performed by using Review manager 5.0, and p < 0.05 was considered statistically significant. The odds ratio (OR) with 95% confidence interval (CI) of CC+CT versus TT genotype of ABCB1 3435C>T polymorphism was pooled for CRC risk. Whether the distributions of genotypes among controls conformed to Hardy-Weinberg equilibrium (HWE) was determined by using a chi-square test and p < 0.05 was considered a departure from HWE. Statistical heterogeneity was measured by using I^2^ statistic and p < 0.05 was considered representative of significant statistical heterogeneity. Heterogeneity was also assessed through visual examination of funnel plots. Fixed effects model was used when there was no heterogeneity of the results of the studies. Otherwise, the random effects model was used. Sensitivity analysis was conducted by omitting each study.

## RESULT

***Characteristics of the included studies: ***There were 8 studies which met the inclusion criteria in this meta-analysis of 2996 cases and 3460 controls in Caucasian (n=7) and Asian (n=1), published between 2005 and 2011. The distributions of genotypes among controls of the whole studies conformed to HWE. The detailed characteristics of the studies included in our meta-analysis are presented in Table-I.

**Fig.1:**
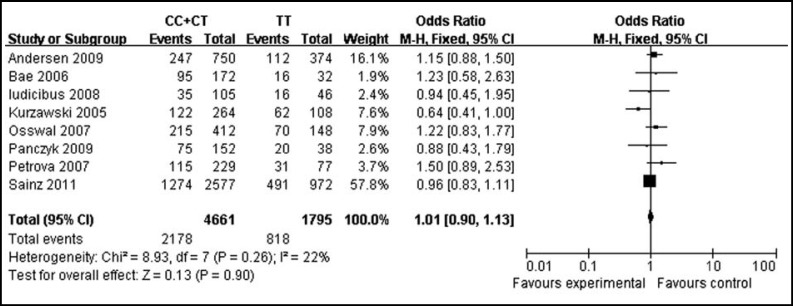
Meta-analysis of the association between the ABCB1 3435C>T polymorphism and CRC risk

**Fig.2:**
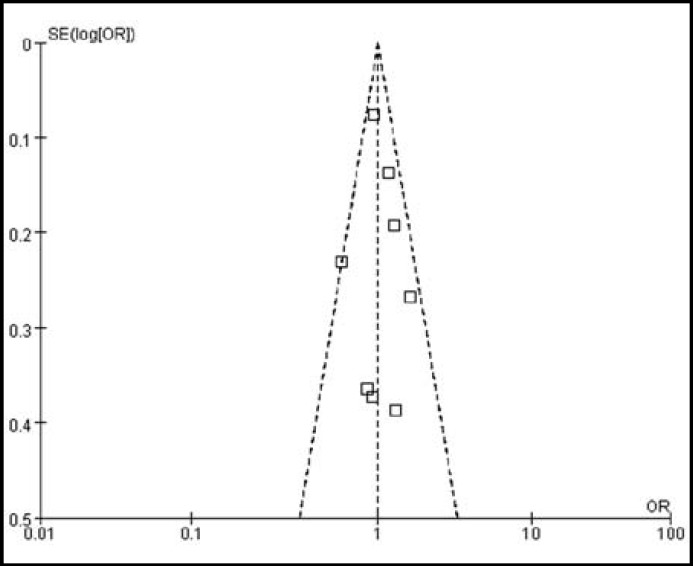
Funnel plots analysis to detect hetero geneity and publication bias

***Association between ABCB1 3435C>T polymorphism and CRC risk: ***It is presented in Fig.1. That there was no significant difference between ABCB1 3435C>T polymorphism and CRC risk in the overall 8 studies (P=0.90). The pooled OR with 95% CI of CC+CT versus TT genotype for CRC risk was 1.01 [0.90-1.13]. There was no evidence of heterogeneity among the studies (I^2^=22%, P=0.26), which was also confirmed through the funnel plots in Fig.2.

We performed a sensitivity analysis which is presented in Table-II, which tested the robustness of the result of the null association between ABCB1 3435C>T polymorphism and CRC risk. Exclusion of individual studies did not modify the estimates much, with pooled ORs ranging from 0.98 to 1.08. The symmetrical shape of the funnel plots which was shown in Fig.2 suggested there was no publication bias among the studies.

**Table-I:** Characteristics of studies included in meta-analysis of ABCB1 3435C>T polymorphism and CRC

*Study*	*Study Population*	*Study Design*	*Genotyping Method*	*Source*	*Case*	*Control*	*HWE*
Panczyk 2009	Polish, Caucasian	Case-control	PCR-RFLP	HB	95	95	P=0.63
Osswald 2007	Russian, Caucasian	Case-control	PCR-RFLP	HB	285	275	P=0.69
Andersen 2009	Danish, Caucasian	Prospective case-cohort	TaqMan, Real-time PCR	PB	359	765	P=0.23
Bae 2006	Korean, Asian	Case-control	PCR-RFLP	HB	111	93	P=0.07
Petrova 2007	Bulgarian, Caucasian	Case-control	PCR-LightCycler	HB	146	160	P=0.16
Sainz 2011	German, Caucasian	Case-control	PCR-KASPar assays	PB	1765	1784	P=0.12
Iudicibus 2008	Italian, Caucasian	Case-control	PCR-RFLP	HB	51	100	P=0.58
Kurzawski 2005	Slavonic, Caucasian	Case-control	PCR-RFLP	HB	184	188	P=1.00

**Table-II:** Sensitivity analysis with each study omitted in fixed-effects model

*Study omitted*	*OR*	*95% CI*	*P*
None	1.01	0.90-1.13	0.90
Andersen 2009	0.98	0.87-1.11	0.75
Bae 2006	1.00	0.90-1.12	0.96
Iudicibus 2008	1.01	0.90-1.13	0.88
Kurzawski 2005	1.04	0.93-1.16	0.53
Osswald 2007	0.99	0.88-1.11	0.86
Panczyk 2009	1.01	0.90-1.13	0.85
Petrova 2007	0.99	0.88-1.11	0.84
Sainz 2011	1.08	0.91-1.27	0.40

## DISCUSSION

This meta-analysis which included 2996 cases and 3460 controls, failed to show an association between ABCB1 3435C>T genotypes and CRC risk.

The ABCB1 gene mainly encodes a plasma membrane pump, P-gp, which protects the cells and organs against from toxic xenobiotic agents and environmental carcinogens by draining various structurally unrelated anticancer agents and toxins.^19^^,^^20^ P-gp was found on apical surfaces of superficial columnar epithelial cells of the colon at high concentrations.^21^ The proposed role of P-gp in the colon is to decrease the deposition of endogenous and exogenous hydrophobic amphipathic toxins^5^^,^^22^ Therefore, the decreased expression or lower activity of P-gp probably contributes to the increased risk of the colon diseases such as CRC and vice versa. Hoffmeyer et al. reported Caucasian individuals with homozygous TT genotype of ABCB1 3435C>T polymorphism had lower intestinal expression of P-gp and greater P-gp substrate digoxin absorption in the intestine after oral administration.^8^

Verstuyft et al. and Johne et al. showed Caucasian individuals with the homozygous TT genotype had higher plasma concentration of digoxin as well.^23^^,^^24^ Hitzl and Drescher et al. also reported Caucasian individuals with the homozygous TT genotype had lower mRNA level of ABCB1 and decreased function of P-gp.^25^^,^^26^ However, the controversy was caused by conflicting studies. Several studies reported Japanese individuals carrying T allele had greater mRNA level of ABCB1 and lower serum concentration of digoxin.^27^^-^^30^ Moreover, Siegmund et al. did not found any influence of ABCB1 3435C>T polymorphism on intestinal expression of P-gp and disposition of P-gp substrate talinolol in Caucasian.^31^ Several studies reported ABCB1 3435C>T polymorphism did not correlate with the expression and function of P-gp in acute myeloid leukemia, recipients of living-donor liver transplantation, peripheral blood lymphocyte and hematopoietic stem cells.^32^^-^^35^

Furthermore, the conflicting data had been noted also for other P-gp substrates such as talinolol, fexofenadine, cyclosporine and tacrolimus.^26^^,^^31^^,^^33^^,^^36^^-^^42^ Possible explanations for those different results are that the 3435C>T polymorphism is a wobble SNP, which may not be the sole SNP affecting the expression of ABCB1 gene and there are the existence of gene-gene interaction and linkage disequilibrium between the 3435C>T polymorphism and other ABCB1 gene polymorphisms. Moreover, the expression of P-gp is probably different in different tissues and the methodology of measuring the expression of P-gp differs between studies. In addition, different substrates of which some are not only mediated by P-gp but also transported by other transmembrane proteins have been used in the various studies with different routes of administration. Thus, there is still no agreement about whether the ABCB1 3435C>T polymorphism has influence on the expression and function of P-gp.

Meanwhile, it also remains controversial that whether the ABCB1 3435C>T polymorphism is associated with CRC risk due to the different results of various studies. Kurzawski et al. reported Caucasian individuals under 50years of age with homozygous TT genotype had the highest CRC risk.^11^ Sainz et al. also found Caucasian males with homozygous TT genotype had higher CRC risk than males carrying C allele.^12^ However, Andersen et al. found Caucasian individuals carrying T allele had lower CRC risk than those with homozygous CC genotype, and individuals with homozygous CC genotype who took red and processed meat were at 8% increased CRC risk.^13^

Osswald et al. reported there was interaction between the combination of several ABCB1 polymorphisms including 3435C>T polymorphism and the status of smoking and age in relation to CRC risk.^14^ Furthermore, several studies did not found association between ABCB1 3435C>T polymorphism and CRC risk.^15^^-^^18^ In addition, Indicibus et al. reported there was no association between the expression of P-gp and ABCB1 3435C>T polymorphism.^18^ The pooled result of this meta-analysis indicated that the homozygous TT genotype of ABCB1 3435C>T polymorphism probably not affect CRC risk. We speculated that ABCB1 3435C>T polymorphism, with other SNPs and factors of sex, age, or lifestyle, probably comes together or partially to affect CRC risk, or perhaps indeed not.

This meta-analysis is potentially limited in the following ways. First, it is difficult to avoid the selection bias since our search was restricted to studies reported in English. Second, not all controls of studies included in our meta-analysis were selected from predominantly healthy populations, and some might have had benign diseases. Therefore, the result should be interpreted with caution because the controls were not uniformly defined. We were also unable to perform subgroup analysis by ethnicity, because there was only one study of Asians and none of Africans. Finally, our meta-analysis was based on unadjusted estimates, and we were unable to perform more precise analysis to examine the interactions of SNPs, age, sex and lifestyle which might be important components of the association between the ABCB1 3435C>T polymorphism and CRC risk because of the lack of individual data.

This meta-analysis provides further evidence that the ABCB1 gene has been highly investigated and plays important roles in CRC, but it is unlikely that the ABCB1 3435C>T polymorphism plays a major role in the etiology of CRC. Therefore, in this context, it is not recommended that individuals are tested for the ABCB1 3435C>T polymorphism as information which is useful in the diagnosis of CRC. Future prospective large well-design study should investigate the influence of the interaction of ABCB1 haplotypes and environment, rather than single SNP so that spurious correlation results are minimized.

## Notes

***Funding:*** No financial support.
